# A web-based data visualization tool for the MIMIC-II database

**DOI:** 10.1186/s12911-016-0256-9

**Published:** 2016-02-04

**Authors:** Joon Lee, Evan Ribey, James R. Wallace

**Affiliations:** School of Public Health and Health Systems, University of Waterloo, Waterloo, ON Canada

**Keywords:** Medical data visualization, Medical data exploration, Web-based software tools

## Abstract

**Background:**

Although MIMIC-II, a public intensive care database, has been recognized as an invaluable resource for many medical researchers worldwide, becoming a proficient MIMIC-II researcher requires knowledge of SQL programming and an understanding of the MIMIC-II database schema. These are challenging requirements especially for health researchers and clinicians who may have limited computer proficiency. In order to overcome this challenge, our objective was to create an interactive, web-based MIMIC-II data visualization tool that first-time MIMIC-II users can easily use to explore the database.

**Results:**

The tool offers two main features: Explore and Compare. The Explore feature enables the user to select a patient cohort within MIMIC-II and visualize the distributions of various administrative, demographic, and clinical variables within the selected cohort. The Compare feature enables the user to select two patient cohorts and visually compare them with respect to a variety of variables. The tool is also helpful to experienced MIMIC-II researchers who can use it to substantially accelerate the cumbersome and time-consuming steps of writing SQL queries and manually visualizing extracted data.

**Conclusions:**

Any interested researcher can use the MIMIC-II data visualization tool for free to quickly and conveniently conduct a preliminary investigation on MIMIC-II with a few mouse clicks. Researchers can also use the tool to learn the characteristics of the MIMIC-II patients. Since it is still impossible to conduct multivariable regression inside the tool, future work includes adding analytics capabilities. Also, the next version of the tool will aim to utilize MIMIC-III which contains more data.

## Background

As many hospitals and clinics across North America and other parts of the world continue to adopt electronic medical record (EMR) systems, research interests have been shifting from EMR data collection to EMR data analytics. This shift in research focus has been fueled by the recent excitement around Big Data analytics and data-driven approaches for clinical decision support and knowledge discovery [1]. In order for medical researchers to advance EMR data analytics, gaining access to large-scale EMR data is the most important first step, and the public intensive care database called MIMIC-II, which has been described by Lee et al. [2], has played an important role in this regard over the past few years. The current public release of MIMIC-II (version 2.6), the descriptive statistics of which are reported in [2], contains rich administrative, demographic, and clinical data from 32,000 unique patients admitted to the intensive care units (ICUs) at Beth Israel Deaconess Medical Center (Boston, MA, USA). More than 725 researchers from more than 32 countries currently have no-cost access to MIMIC-II (upon completing human subjects training and signing a data use agreement), and the number of MIMIC-II users continues to grow by over 50 % per year. Numerous research studies have been published based on MIMIC-II [3–6].

Approved MIMIC-II users can access the clinical part of the database (as opposed to the waveforms, i.e., physiologic tracings such as electrocardiograms gathered from ICU bedside monitors, which can be accessed through other methods described on PhysioNet [7]) via two most popular ways [8]: 1) the web-based tool called QueryBuilder or 2) a MIMIC-II virtual machine (VM). There is also a third, less frequently used option where the user can download the raw data in text files and use a schema builder to manually construct the database. Although both QueryBuilder and the VM are easy-to-use and platform-independent, they require knowledge in Structured Query Language (SQL) which has been identified as the biggest barrier for clinicians and health researchers who may lack the relevant computer programming skills. Even for computer scientists and engineers well-versed in SQL, there is a non-trivial learning curve associated with understanding the MIMIC-II database schema as well as the clinical nuances involved in understanding EMR data.

While comprehensive medical data warehousing and analytics platforms such as STRIDE [9] and i2b2 [10] exist, public web-based tools for convenient data visualization and exploration have been almost non-existent in medical research, not to mention that truly public medical databases have been rare as well. MIMIC-II already is an innovation in that it is available to any interested researcher for free, but the impact of MIMIC-II can be further extended by going beyond simply granting access and making it easy for any medical researcher to rapidly explore MIMIC-II.

To facilitate and accelerate initial steps for anyone wishing to utilize MIMIC-II for their research, our objective was to develop a web-based MIMIC-II data visualization tool that would enable a quick, convenient MIMIC-II data exploration for preliminary investigation and feasibility estimation prior to embarking on a research study. The purpose of the tool is to allow the user to quickly visualize key aggregate statistics (e.g., sample size, distributions of clinical variables) of a patient cohort of interest within MIMIC-II, rather than to enable the user to download raw patient-level data, which should still be accomplished via QueryBuilder or the MIMIC-II VM.

## Implementation

### Main visualization features

The MIMIC-II data visualization tool was designed to provide two main features: Explore and Compare. The Explore feature was implemented in such a way that the user can select a patient cohort based on admission ICU service type, gender, age, and primary International Classification of Diseases 9 (ICD-9) code. The four adult ICU service types in MIMIC-II are medical ICU (MICU), surgical ICU (SICU), coronary care unit (CCU), and cardiac surgery recovery unit (CSRU), with a total of 24,538 unique patients from these units. The fifth ICU service type in MIMIC-II is neonatal ICU (NICU) which was excluded from this tool’s scope due to neonates’ physiologic and clinical differences compared to adults. Then, the user can select the variables to be visualized within the selected cohort from categorized lists, which are tabulated in Table 1. For vital signs and lab test results, the user can select from the following five options as well: the admission, discharge, minimum, maximum, or average value of the ICU stay. Once the tool is finished plotting the results, continuous and categorical variables are displayed in histograms and pie charts, respectively, except the total numbers of unique ICU stays and patients, patient outcomes, and interventions, which are shown in text.

**Table 1:** Categories and their corresponding variables that the user can select to visualize

Category	Variables
Demographic information	age, ethnicity, gender, marital status, religion
Administrative information	admission ICU service type, admission source, admission type, number of days between ICU and hospital admissions, insurance type
Patient outcomes	mortality at 28 days post-discharge from the hospital, hospital length of stay, hospital mortality, ICU mortality, ICU length of stay, 2-year survival days post-discharge from the hospital
Vital signs	heart rate, mean arterial blood pressure, oxygen saturation, respiratory rate, systolic blood pressure, body temperature
Lab test results	bicarbonate, calcium, chlorine, creatinine, glucose, hematocrit, lactate, magnesium, phosphorus, potassium, sodium, white blood cell count
Interventions	hemodialysis, peritoneal dialysis, total time on mechanical ventilation, vasopressor administration
Miscellaneous	amount of colloids administered, amount of crystalloids administered, body mass index, fluid balance, height, primary ICD-9, SOFA (sequential organ failure assessment) score, SAPS (simplified acute physiology score) I score, urine output, weight

The Compare feature was designed to enable the user to visually compare two patient cohorts with respect to selected variables to be visualized. For both cohorts, the same cohort selection criteria as those available in the Explore feature are available. The categorized variables in Table 1 are again available for visualization in the Compare feature. For comparison purposes, continuous and categorical variables are visualized in box plots and stacked bar plots, respectively. Like the Explore feature, the following information is displayed in text: the total numbers of unique ICU stays and patients, patient outcomes, and interventions.

Both the Explore and Compare features were designed to exclude missing data from visualization.

### Front-end and back-end of the tool

The front-end of the MIMIC-II data visualization tool was designed to be a web-based graphical user interface (GUI) that can be accessed from any web browser. The front-end at the client side communicates with an Apache web server hosted in the Health Data Science Lab (HDSL) at the University of Waterloo. In turn, this web server submits a data request to a database server in the HDSL that hosts a copy of MIMIC-II. Once the retrieved data are reformatted and relayed back to the user by the web server, the visualization results are further processed and displayed within the client’s web browser. Overall, the tool incorporates multiple programming languages. The front-end was created using HTML, CSS, and the JavaScript libraries jQuery and D3.js. The back-end (the server side) of the tool was programmed in PHP and PostgreSQL.

**Fig. 1:**
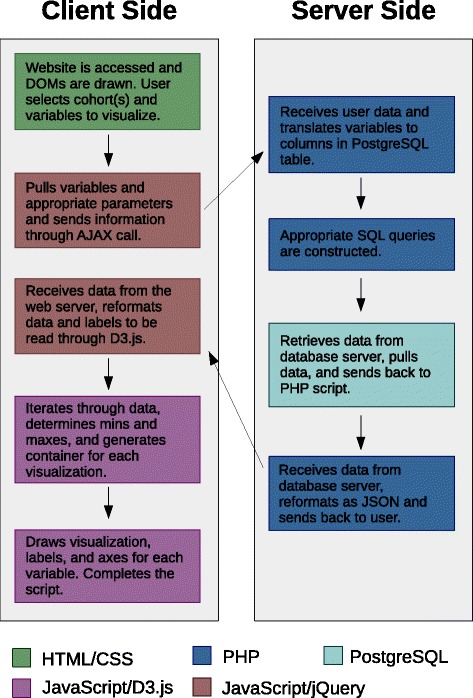
A diagram describing how the visualization tool works. The sequence of steps is indicated by arrows, while the colour of each box refers to the programming language used

For the ensuing detailed description of the implementation, refer to Fig. 1 for an easy-to-understand illustration. The tool starts by generating the static portion of the webpage by pulling the appropriate information files from the web server. Upon user selection of cohort criteria and variables to be visualized, the required information is pulled from each of the appropriate document object models (DOMs) through jQuery, stored in a JSON object, and sent to the web server through an Asynchronous JavaScript and XML (AJAX) call. The information is sent through an AJAX call as opposed to page forwarding in order to store previous results and pull information from different tabs on the GUI upon switching between the Explore and Compare features.

Once the client’s web browser sends a request to the web server, a PHP script filters and cross-references the information from the user through a dictionary that translates the requested variables to their corresponding columns in a custom PostgreSQL table. This table is not part of the standard MIMIC-II release and essentially contains all ICU stays as rows and all variables for cohort selection and visualization as columns. This table was created based on multiple tables contained in the original MIMIC-II schema so that computational time can be minimized on the fly by bypassing computationally expensive table joins. Subsequently, an appropriate PostgreSQL query is generated and submitted to the database server to extract the required data. Separate queries are constructed and submitted for the results that are displayed in text later (i.e., the total numbers of unique ICU stays and patients, patient outcomes, and interventions). Once all data extraction and processing is completed on the server side, the final data are sent back to the user as a JSON object. Additional information that is included within the JSON object are variable names and units of measurement.

Once the data arrive at the client side, D3.js-based scripts process and reformat the received JSON object so that it can be interpreted by D3.js, while differentiating continuous and categorical variables. D3.js then iterates over the variables to be visualized and draws an appropriate scalar vector graphic (SVG) for each. Finally, titles, axes, and labels are added to the figures, and the visualization is ready for viewing on the user’s web browser.

## Results and Discussion

The MIMIC-II data visualization tool is available at http://hdsl.uwaterloo.ca/visualization-tool/. The GUI allows the user to toggle among three tabs: Introduction, Explore, and Compare. The Introduction tab displays general information about the tool, MIMIC-II, and the creators of the tool. The Explore and Compare tabs present their respective features. On all three tabs, a disclaimer for the user is stated at the bottom of the webpage.

### The Explore feature

**Fig. 2:**
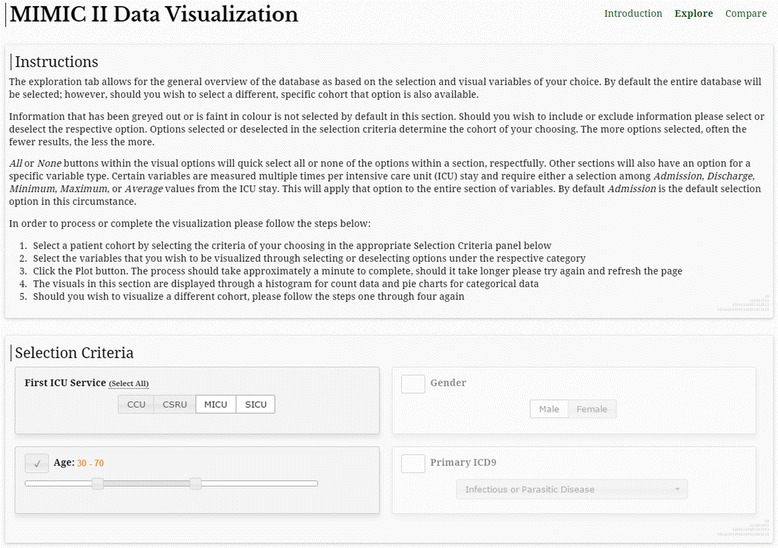
A screenshot of the visualization tool showing the Explore feature. Selection of all MICU and SICU patients aged between 30 and 70 is shown

Figure 2 shows a screenshot of the Explore tab. Instructions for how to use the Explore feature is shown at the top of the webpage. In the Selection Criteria panel located immediately below the Instructions panel, the user can set cohort inclusion and exclusion criteria.

**Fig. 3:**
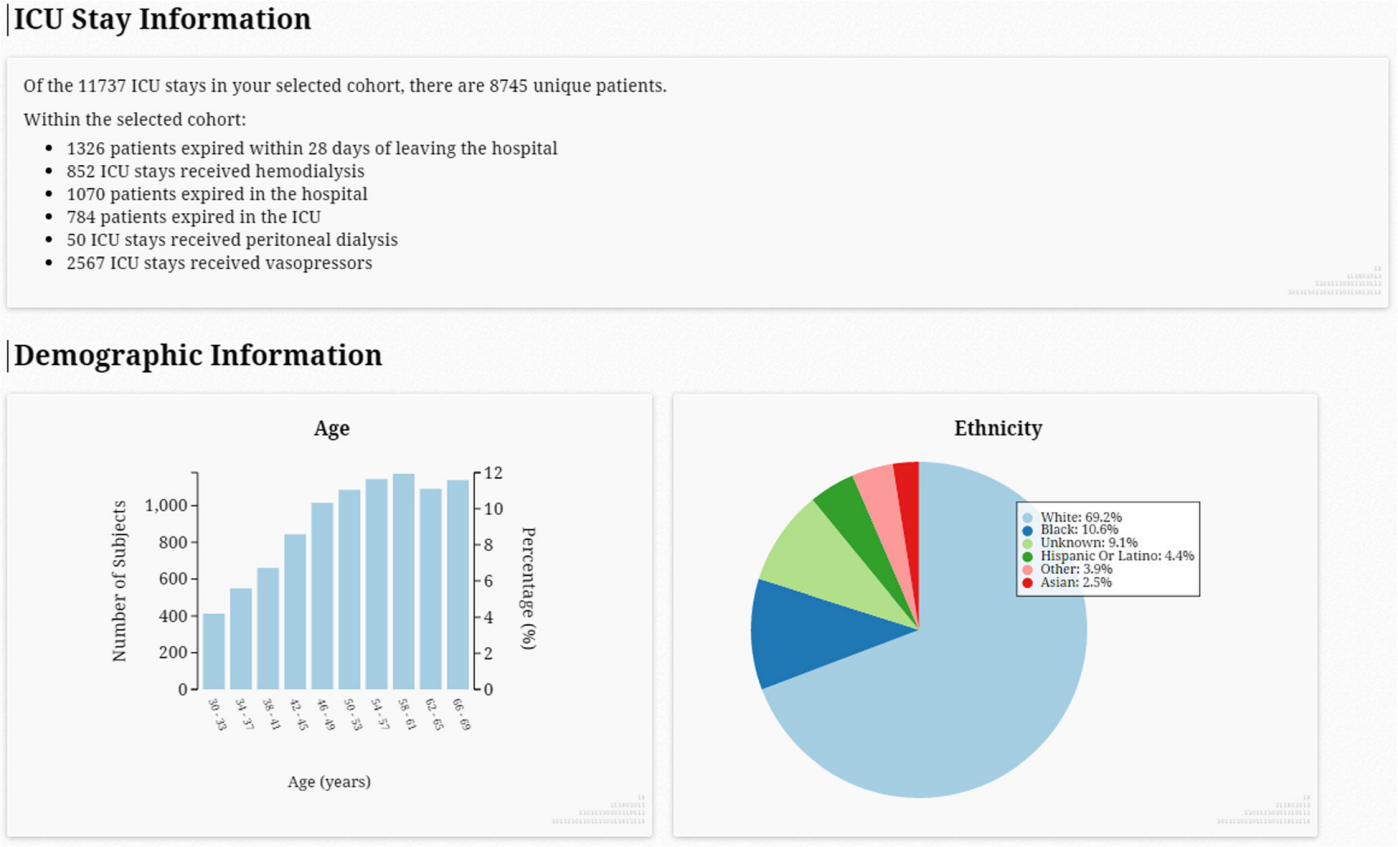
A screenshot of an example Explore visualization. The results correspond to the cohort selection in Fig. 2

Figure 3 shows part of the visualization results for the cohort selection criteria shown in Fig. 2. The numbers of unique patients and ICU stays included in the selected cohort as well as intervention and patient outcome information are displayed textually in the ICU Stay Information panel. Under Demographic Information in Fig. 3, the two types of visualization, namely histogram and pie chart, are demonstrated for age and ethnicity. All other continuous and categorical variables are visualized in similar histograms and pie charts, respectively.

### The Compare feature

**Fig. 4:**
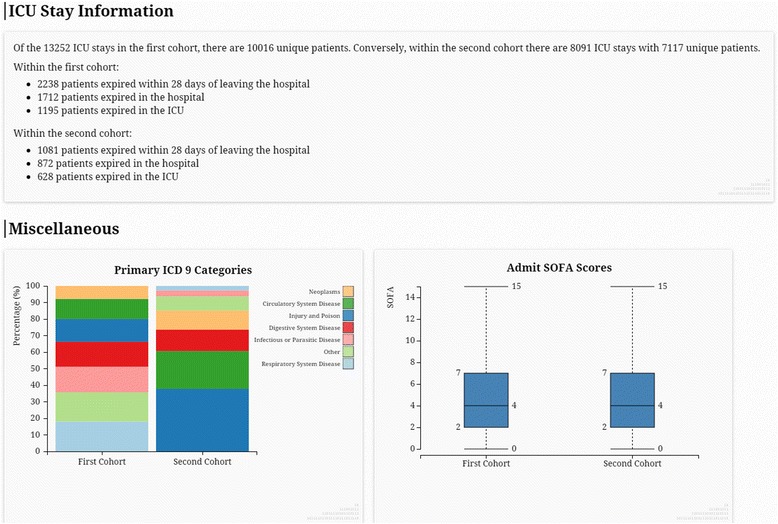
A screenshot of an example Compare visualization. The first and second cohorts selected here are the entire MICU and SICU cohorts, respectively. Only the mortality outcomes (28-day, hospital, and ICU mortality), primary ICD-9, and admission SOFA score were selected to be visualized

The top of the Compare tab resembles Fig. 2, except that the instructions are slightly different and that there are two cohort selection panels (each is identical to the Selection Criteria panel shown in Fig. 2). As an example, Fig. 4 shows the Compare results when the first and second cohorts are the entire MICU and SICU patients, respectively, and the following variables are selected to be visualized: the three mortality outcomes (28-day, hospital, and ICU mortality), primary ICD-9, and admission SOFA. Since ICD-9 and SOFA are categorical and continuous, respectively, they are visualized as a stacked bar plot and a box plot, respectively.

### Possible applications

The MIMIC-II data visualization tool provides a convenient way to quickly explore MIMIC-II prior to embarking on a research study. For researchers who are new to MIMIC-II, the tool enables them to conduct a preliminary investigation on MIMIC-II without any knowledge of SQL or the MIMIC-II database schema. Such a preliminary investigation often targets checking sample size and descriptive statistics prior to an in-depth research study, or simply learning the characteristics of the MIMIC-II patients by studying the distributions of various variables. Also, the Compare feature of the tool allows researchers to visually check for unadjusted differences between patient cohorts with respect to selected variables.

For example, the Explore example shown in Figs. 2 and 3 can be used to see if sample size is sufficiently large to study MICU and SICU patients aged between 30 and 70 who underwent peritoneal dialysis. Figure 3 shows that there are only 50 ICU stays that meet the inclusion criteria, which would be considered too small for most multivariable regression models. Furthermore, the Compare example shown in Fig. 4 can be conducted to check feasibility for mortality studies comparing MICU and SICU. The difference in crude mortality rates between MICU and SICU can readily be calculated, and the differences in ICD-9 codes and SOFA scores can help with understanding the overall severity of illness and patient characteristics in the two cohorts. These observations can inform selection of confounding variables.

The tool is also helpful to experienced researchers who are already familiar with MIMIC-II, since it allows them to bypass the cumbersome steps of 1) writing and running SQL queries, and 2) loading, processing, and analyzing the extracted data in an analytics environment (e.g., R, Python). Iterating through these steps are inevitable at the beginning of most research projects that involve MIMIC-II [8], especially when various patient sub-cohorts within MIMIC-II need to be checked for study feasibility. By substantially speeding up the time-consuming steps described above, the tool is valuable even to experienced researchers who can use the tool to quickly and efficiently screen for feasibility of research ideas.

### Limitations

The most prominent limitation of the MIMIC-II data visualization tool is that it can only aid simple data exploration. Once the researcher is content with the outcome of a preliminary investigation based on the tool, the core of most research studies, such as multivariable regression, usually requires an elaborate SQL query for extraction of raw patient-level, rather than aggregate, data. At this stage, the researcher is required to understand the MIMIC-II database schema and run the SQL query using either QueryBuilder or the MIMIC-II VM.

Another limitation is that the tool can take up to a minute to complete a given visualization job, especially when all available variables are selected for visualization. The running time can be substantially shortened by either deselecting variables that the user does not wish to visualize or by using a more powerful computer at the client side, since the most time-consuming step is the iterations through all selected variables for SVG preparation at the client side.

Given that health services delivery and medical technologies constantly change over time, an important dimension for MIMIC-II researchers is date of ICU admission. However, all actual dates in MIMIC-II have been randomly shifted through a de-identification process in order to comply with the Health Insurance Portability and Accountability Act (HIPAA) for public release [2]. Hence, an important limitation of MIMIC-II, and of the MIMIC-II data visualization tool as a result, is that it is impossible to study trends or changes over time.

Lastly, because NICU data were excluded from the tool, researchers interested in neonatal intensive care cannot benefit from the tool.

### Future work

To address the lack of analytics described in the Limitations section above, a promising future direction is to add a third feature to the tool, namely Analyze. The Analyze feature would allow the user to conduct simple statistical tests such as a *t*-test or a chi-square test, as well as multivariable logistic regression using user-selected covariates and patient outcomes within a specific cohort, all at the convenience of a few mouse clicks within a web browser. Furthermore, enabling the user to develop machine learning algorithms as part of the Analyze feature is a possibility. Since JavaScript offers limited analytics functionalities, it may be best to implement the Analyze feature in R, Python, or Java.

In addition, an export function seems desirable in the next version of the tool. Such a function would enable the user to export individual figures or raw data that led to the figures. Since only approved MIMIC-II users should be able to download raw patient-level data due to the need for human subjects training and a data use agreement, the user would be asked to log in using their official MIMIC-II account before using the data download function.

The provider order entry (POE) data in MIMIC-II contain useful information about prescribed medications. However, these data require careful pre-processing since both generic and branded medication names are used interchangeably. The algorithms developed by Warner et al. [11] can be incorporated by the MIMIC-II data visualization tool to normalize and show medication data.

Lastly, the latest version of MIMIC, MIMIC-III, was released to the public in August 2015 (see http://mimic.physionet.org). MIMIC-III contains approximately 46,000 unique patients, including all MIMIC-II patients. A meaningful future step would be to adapt the visualization tool to MIMIC-III, although this would be more complicated than simply having the tool point to MIMIC-III due to the major differences between MIMIC-II and MIMIC-III schemas. Furthermore, including all NICU data in MIMIC-III would be another way to expand the utility of the tool.

## Conclusions

The MIMIC-II data visualization tool is an intuitive, interactive web-based tool that allows any researcher to explore MIMIC-II without any knowledge of SQL or the MIMIC-II database schema. It provides the flexibility of selecting patient cohorts of interest, as well as a long list of administrative, demographic, and clinical variables that can be visualized. This tool can greatly facilitate and accelerate the preliminary investigation stage of MIMIC-II-based research studies, for both experienced and inexperienced MIMIC-II researchers.

### Availability and requirements

- **Project name**: MIMIC-II data visualization
- **Project home page**: http://hdsl.uwaterloo.ca/visualization-tool/
- **Operating system** (**s**): Platform independent
- **Programming language**: JavaScript, D3.js, jQuery, PHP, PostgreSQL, HTML, CSS
- **Other requirements**: Any web browser
- **License**: Free to use, source code available upon request
- **Any restrictions to use by non**-**academics**: None

## Abbreviations

AJAX: asynchronous JavaScript and XML
CCU: coronary care unit
CSRU: cardiac surgery recovery unit
DOM: document object model
EMR: electronic medical record
GUI: graphical user interface
ICD: international classification of diseases
ICU: intensive care unit
MICU: medical intensive care unit
SICU: surgical intensive care unit
SQL: structured query language
SVG: scalar vector graphic
VM: virtual machine
